# Evaluation of the Efficacy and Safety of Rivaroxaban Using a Computer Model for Blood Coagulation

**DOI:** 10.1371/journal.pone.0017626

**Published:** 2011-04-22

**Authors:** Rolf Burghaus, Katrin Coboeken, Thomas Gaub, Lars Kuepfer, Anke Sensse, Hans-Ulrich Siegmund, Wolfgang Weiss, Wolfgang Mueck, Joerg Lippert

**Affiliations:** 1 Bayer Schering Pharma AG, Wuppertal, Germany; 2 Bayer Technology Services GmbH, Leverkusen, Germany; 3 Bayer Schering Pharma AG, Berlin, Germany; University of South Florida College of Medicine, United States of America

## Abstract

Rivaroxaban is an oral, direct Factor Xa inhibitor approved in the European Union and several other countries for the prevention of venous thromboembolism in adult patients undergoing elective hip or knee replacement surgery and is in advanced clinical development for the treatment of thromboembolic disorders. Its mechanism of action is antithrombin independent and differs from that of other anticoagulants, such as warfarin (a vitamin K antagonist), enoxaparin (an indirect thrombin/Factor Xa inhibitor) and dabigatran (a direct thrombin inhibitor). A blood coagulation computer model has been developed, based on several published models and preclinical and clinical data. Unlike previous models, the current model takes into account both the intrinsic and extrinsic pathways of the coagulation cascade, and possesses some unique features, including a blood flow component and a portfolio of drug action mechanisms. This study aimed to use the model to compare the mechanism of action of rivaroxaban with that of warfarin, and to evaluate the efficacy and safety of different rivaroxaban doses with other anticoagulants included in the model. Rather than reproducing known standard clinical measurements, such as the prothrombin time and activated partial thromboplastin time clotting tests, the anticoagulant benchmarking was based on a simulation of physiologically plausible clotting scenarios. Compared with warfarin, rivaroxaban showed a favourable sensitivity for tissue factor concentration inducing clotting, and a steep concentration–effect relationship, rapidly flattening towards higher inhibitor concentrations, both suggesting a broad therapeutic window. The predicted dosing window is highly accordant with the final dose recommendation based upon extensive clinical studies.

## Introduction

**Figure 1:**
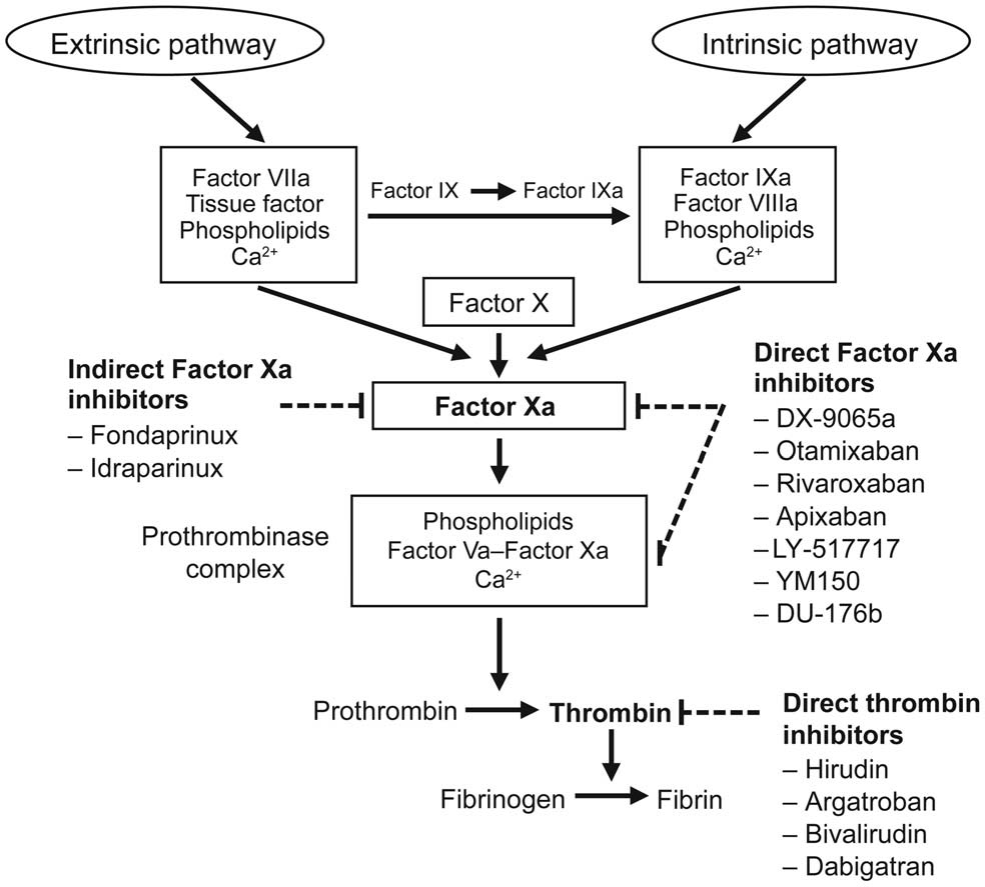
Targets for anticoagulant drugs in the coagulation pathway [**57**].

The blood coagulation cascade is a complex process, involving both an intrinsic and an extrinsic pathway (Figure 1) [1]. The different classes of anticoagulant drugs currently on the market or in clinical development target different factors within the coagulation cascade (Figure 1). The most interesting new classes of anticoagulants include the direct thrombin inhibitors and the Factor Xa inhibitors (direct or indirect).

Rivaroxaban is an oral, direct Factor Xa inhibitor approved in more than 100 countries worldwide, including the European Union and Canada, for the prevention of venous thromboembolism after elective hip or knee replacement surgery in adult patients, and is in advanced clinical development for the treatment of thromboembolic disorders. Rivaroxaban only targets one factor within the coagulation cascade, Factor Xa, and its mechanism of action is antithrombin (AT) independent [2], [3]. This mechanism of action is different from that of other anticoagulants that have been or are currently used in clinical practice, such as warfarin (a vitamin K antagonist) [4], enoxaparin (an indirect thrombin/Factor Xa inhibitor) [5], [6], ximelagatran (now withdrawn) [7] and dabigatran (direct thrombin inhibitors) [8].

A computer model for blood coagulation has been developed, based on several published models [9]–[14]. In contrast to these previous models, the one presented here takes into account both the intrinsic and extrinsic pathways of the coagulation cascade and possesses some unique features that were not included in these earlier models, such as a blood flow component and a portfolio of drug action mechanisms based on their physicochemical properties and pharmacokinetic profiles. The aim of this study was to use this model to evaluate the efficacy and safety of different doses of rivaroxaban compared with other anticoagulants, thereby estimating a therapeutic window for rivaroxaban.

The model was built to cover several aspects of the coagulation cascade, including the extrinsic pathway (initiated with the triggering of tissue factor [TF]), the intrinsic pathway (initiated with activation of Factor XII [Factor XIIa]) and the common pathways leading to fibrin generation via thrombin generation. Additional features were also included: inhibition via the TF pathway inhibitor or via AT, and the fact that *in vivo* coagulation is affected by blood flow, which leads to an exchange of proteins between the clot and the fresh blood pool. The action of calcium ions (within membrane-bound enzyme complexes, e.g. the prothrombinase complex) were indirectly included in the rate constants and kinetic parameters, and phospholipid membrane-binding sites were directly included in the model kinetics. A portfolio of drug action mechanisms and pharmacokinetic profiles was also included in the model: rivaroxaban and DX-9065a (direct Factor Xa inhibitors), warfarin, enoxaparin and ximelagatran. The model was adjusted to accurately represent both preclinical information, such as directly measured dissociation constants, and clinical measurements such as the common clotting tests, prothrombin time (PT) and activated partial thromboplastin time (aPTT). Some additional aspects of the coagulation cascade will be developed in further improvements to the model, including: thrombocytes and their action; fibrinolysis and spatial thrombus properties.

This computer model was used to assess the efficacy and safety of rivaroxaban compared with that of other anticoagulants included in the model, by using physiologically plausible clotting scenarios with presumably high relevance for bleeding as well as thrombosis events. The computer model was subsequently applied to the modelling of the therapeutic window of rivaroxaban by comparing the inhibitory effect of all anticoagulants in these virtual clotting scenarios, which were not feasible with laboratory *in vitro* test systems.

## Methods

### Structural properties of the model

The program package MoBi® (Bayer Technology Services, Leverkusen, Germany) [15] was used as the software platform. The structural properties of the model represent qualitative knowledge about the coagulation processes. This is exemplified by the cleavage and activation of prothrombin (Factor II) to thrombin (Factor IIa) by Factor Xa. This reaction is included in the model to take into account both the stoichiometric part:
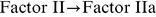
(1)and the kinetics part:
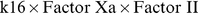
(2)(see: Appendix S1, Reaction R9)

Equation 1 represents the one-to-one (stoichiometric) conversion of Factor II to Factor IIa (prothrombin to thrombin). Equation 2 describes the kinetic law governing the rate of conversion: it is proportional to the concentrations of Factor Xa, Factor II, with k16 being the kinetic constant. Kinetic parameters and initial conditions (concentrations) represent the quantitative information about the coagulation system and take into account the impact of the different study drugs (see: Appendix S2 and Appendix S3). Kinetic equations are given according to standard mass action law convention.

### The coagulation model

Individual factors and interactions were classified using the well-characterized extrinsic or intrinsic pathways, because the model was constructed to represent the typical in vitro tests used for investigating the extrinsic and intrinsic processes (PT and aPTT, respectively) in one unified computational representation. Transport reactions taking into account the blood flow were then incorporated, so that the model was more closely related to the physiological in vivo setting.

The extrinsic core of the model was adapted from the model by Hockin et al. [10] and consists of 32 reactions (see: Appendix S1, reactions named with R and number). It covers all relevant interactions and factors, from the triggering of the cascade by TF, to Factor II (prothrombin) activation and Factor IIa (thrombin) formation. The PT test best assesses coagulation activity in the extrinsic pathway.

The intrinsic pathway was adapted from the model published by Kogan et al. [11] and consists of the reactions listed in Appendix S1 containing rate constants starting with ‘Kog’ (at the end of the appendix). It covers all relevant interactions and factors leading from Factor XIIa to Factor Xa. The aPTT test best assesses coagulation activity in the intrinsic pathway.

Further model extensions were taken from the model developed by Anand et al. [14]. A feedback loop for the activation of Factor XI and a reaction representing the cleavage of fibrinogen (RI), also introduced by Kogan et al. [11], [16] and kinetic data taken from Stevens et al. [16] were added to the original model to define two independent thresholds for thrombus formation – one based on Factor IIa and the other on the fibrinogen cleavage product (named ‘Ia’ in the model and being used as a representation of fibrin formation) concentration, respectively.

The protein C/S system and the coagulation factor adsorption reactions to lipids were developed on the basis of the model published by Bungay et al. [9] and by Kuharsky and Fogelson [13]. In addition to reactions published by these authors, the protein C/S model part was extended by us with further reactions that reflect additional inactivation paths (see: Appendix S1, reaction names starting with ‘RBu’ containing extra letters appended to the number). The integration of phospholipid vesicles led to a set of adsorption reactions (see: Appendix S1, reaction names starting with ‘Rad’), but we also needed to duplicate several reactions to account for the option that several of the components might react either when bound to phospholipids or when being dissolved (see: Appendix S1, reaction names of the scheme ‘R’ number ‘d’). The species ‘PhosphoLipid’ represents protein-binding sites on phospholipid vesicles. Bungay et al. use an average of 100 phospholipid molecules per site, but our model was set to 333 molecules per site, being of the same size as published experimental values [17].

Additional coagulation factor inhibition reactions were introduced based on published rate constants [18]–[23]. The rate constant for the inhibitor AT (ATIII), which was in the upper region of experimentally obtained values in the model of Hockin et al. [10], was reduced in our model. The action of von Willebrand factor was introduced based on the scheme and rate constants published by Saenko et al. [24].

### Drug action

All study drugs were modelled by a representation of their anticoagulant properties using what was known from published information. Values for direct kinetic constants (k_on_ and k_off_) were used when available. When no direct experimental kinetic data for individual biochemical reactions were available, values were determined numerically by fitting the model behaviour to indirect measurements using PT and aPTT values versus drug concentrations. A broad set of regimens (doses and schedules) was simulated, both with constant drug levels and dynamic pharmacokinetic profiles (Table 1).

**Table 1:** Regimens for rivaroxaban, warfarin, ximelagatran, DX-9065a and enoxaparin included in the model.

Rivaroxaban		Warfarin		Ximelagatran		DX-9065a		Enoxaparin	
Dose (mg)	Plasma level (µg/l)	Dose (INR)	Plasma level	Dose (mg)	Plasma level (µM)	Dose	Plasma level (µg/l)	Dose (mg s.c.)	Plasma level (mg/l)
5 OD	C_max_ 60.98	1.5		24 BD	C_max_ 0.21	IV infusion	100	20 OD	C_max_ 1.79
	C_mean_ 24.280	2.0			C_mean_ 0.12		200		C_mean_ 0.76
	C_trough_ 4.27	2.5			C_trough_ 0.04				C_trough_ 0.19
5 BD	C_max_ 75.97	3.0		60 BD	C_max_ 0.52			40 OD	C_max_ 3.69
	C_mean_ 42.83	3.5			C_mean_ 0.31				C_mean_ 1.57
	C_trough_ 16.36	4.0			C_trough_ 0.12				C_trough_ 0.40
10 OD	C_max_ 121.97							30 BD	C_max_ 2.74
	C_mean_ 48.56								C_mean_ 1.95
	C_trough_ 8.54								C_trough_ 0.95
10 BD	C_max_ 151.9							105 OD (1.5 mg/kg)	C_max_ 12.8
	C_mean_ 85.66								C_mean_ 5.47
	C_trough_ 32.73								C_trough_ 1.40
20 OD	C_max_ 195.15							70 BD (1 mg/kg)	C_max_ 10.79
	C_mean_ 77.69								C_mean_ 7.67
	C_trough_ 13.66								C_trough_ 3.74
20 BD	C_max_ 243.10								
	C_mean_ 137.06								
	C_trough_ 52.37								
53 OD	C_max_ 387.86								
	C_mean_ 154.42								
	C_trough_ 27.15								
53 BD	C_max_ 483.17								
	C_mean_ 272.42								
	C_trough_ 104.08								

53 mg OD/BD doses (although not used in clinical studies) were simulated to double exposure (C_mean_) of 20 mg OD/BD, taking into account the less than dose-proportional increase in exposure due to reduced absorption, observed at high doses [3], [26]. A control without any drugs was also run in the model. BD, twice daily; C_max_, maximum drug concentration; C_mean_, average drug concentration; C_trough_, minimum drug concentration; INR, international normalized ratio; IV, intravenous; OD, once daily; s.c., subcutaneous.

#### Rivaroxaban

Rivaroxaban acts via complexation to free Factor Xa and to the prothrombinase complex (Factor Xa and activated Factor V [Factor Va]; Table 2) [2]. Experimental kinetic data were available for the reaction of complexation to Factor Xa [25]. Kinetic constants were calculated and fitted into the model based on measured Ki values [2] and experimental PT–rivaroxaban plasma concentration relationships [26]. The binding of rivaroxaban to proteins was modelled as a complexation/decomplexation reaction (RBay3), and kinetic constants were calculated and fitted into the model based on the values of measured fraction of unbound drug and PT versus rivaroxaban plasma concentration relationships. This method was used to model protein binding for all other study drugs, with some variations to take the different mechanisms of action into account.

**Table 2:** Modelling of drug action.

Reaction name	Stoichiometry	Kinetics
**Rivaroxaban action**		
RBay1	Bay59_7939+Xa→Bay59_7939_Xa	Bay59_7939*Xa*kBay1-Bay59_7939_Xa*kBay1*kBay_Ki_Xa
RBay1s	Bay59_7939+Xa_lipid→Bay59_7939_Xa_lipid	Bay59_7939*Xa_lipid*kBay1-Bay59_7939_Xa_lipid*kBay1*kBay_Ki_Xa
RBay2	Bay59_7939+Xa_Va_lipid→Bay59_7939_Xa_Va_lipid	Bay59_7939*Xa_Va_lipid*kBay3-Bay59_7939_Xa_Va_lipid*kBay3*kBay_Ki_XaVa
RBay3	Bay59_7939_Bound→Bay59_7939	kBay_fu_on*kBay_fu*Bay59_7939_Bound-kBay_fu_on*Albumin_Factor*Bay59_7939
RBay4	Bay59_7939_Xa+ATIII→Bay59_7939_Xa_ATIII	kBay5*Bay59_7939_Xa*ATIII
RBay5	Bay59_7939+Xa_ATIII→Bay59_7939_Xa_ATIII	kBay6*Bay59_7939*Xa_ATIII-kBay6*kBay_Ki_XaATIII*Bay59_7939_Xa_ATIII
**DX-9065a action**		
RDx1	DX9065a+Xa→DX9065a_Xa	kDx1*DX9065a*Xa-kDx2*DX9065a_Xa
RDx1s	DX9065a+Xa_lipid→DX9065a_Xa_lipid	kDx1*DX9065a*Xa_lipid-kDx2*DX9065a_Xa_lipid
RDx2	DX9065a+Xa_Va_lipid→DX9065a_Xa_Va_lipid	kDx3*DX9065a*Xa_Va_lipid-kDx4*DX9065a_Xa_Va_lipid
RDx3	DX9065a_Xa+ATIII→DX9065a_Xa_ATIII	kDx5*DX9065a_Xa*ATIII
RDx4	DX9065a→Dx9065a_Bound	kDx_fu_on*Albumin_Factor*Dx9065a-kDx_fu_on*kDx_fu*DX9065a_Bound
RDx5	DX9065a+Xa_ATIII→DX9065a_Xa_ATIII	kDx6*DX9065a*Xa_ATIII-kDx7*DX9065a_Xa_ATIII
**(Xi)Melagatran action**		
RXi1	Xim+IIa→IIa_Xim	kXim1*IIa*Xim-kXim2*IIa_Xim
RXi2	Xim+mIIa→mIIa_Xim	kXim3*mIIa*Xim-kXim4*mIIa_Xim
RXi3	Xim+IIa_Tm→IIa_Tm_Xim	kXim5*IIa_Tm*Xim-kXim6*IIa_Tm_Xim
RXi4	Xim→Xim_Bound	kXim_fu_on*Albumin_Factor*Xim-kXim_fu_on*kXim_fu*Xim_Bound
**Enoxaparin action**		
RHep1	Hep→Hep_Bound	kHep_fu_on*Albumin_Factor*Hep-kHep_fu_on*kHep_fu*Hep_Bound
RHep2	ATIII+Hep→ATIIIa	kHep_ATIII_on*ATIII*Hep-kHep_ATIII_on*kHep_Ki_ATIII*ATIIIa
RHep3	Xa+ATIIIa→Xa_ATIII+Hep	kHep_Xa_ATIIIa*Xa*ATIIIa
RHep4	Xa_lipid+ATIIIa→Xa_ATIII+PhosphoLipid+Hep	kHep_Xa_ATIIIa*Xa_lipid*ATIIIa
RHep5	Xa_Va_lipid+ATIIIa→Xa_ATIII+Hep+Va_lipid	kHep_XaVa_ATIIIa*Xa_Va_lipid*ATIIIa
RHep6	IIa+ATIIIa→IIa_ATIII+Hep	kHep_IIa_ATIIIa*IIa*ATIIIa
RHep7	mIIa+ATIIIa→mIIa_ATIII+Hep	kHep_IIa_ATIIIa*mIIa*ATIIIa
RHep8	ATIIIa+IXa→IXa_ATIII+Hep	kHep_IXa_ATIIIa*IXa*ATIIIa
RHep9	ATIIIa+IXa_lipid→IXa_ATIII+PhosphoLipid+Hep	kHep_IXa_ATIIIa*IXa_lipid*ATIIIa
RHep10	XIa+ATIIIa→XIa_ATIII+Hep	kHep_XIa_ATIIIa*XIa*ATIIIa
RHep11	XIa_lipid+ATIIIa→XIa_ATIII+PhosphoLipid+Hep	kHep_XIa_ATIIIa*XIa_lipid*ATIIIa

Va, IIa, IXa, Xa and XIa denote activated coagulation factors. ATIII, antithrombin; BAY59-7939, rivaroxaban; fu, fraction unbound; Hep, heparin (here parameterized as enoxaparin); on, k_on_, association rate constant; mIIa, meizothrombin; Tm, thrombomodulin; Xim, ximelagatran active metabolite (melagatran).

#### DX-9065a

The method used to fit DX-9065a into the model was similar to that used for rivaroxaban, because the two drugs have a similar mechanism of action [27]. Because DX-9065a–Factor Xa complexes can bind Factor Va, this reaction was added to the model (RDx3; Table 2). All kinetic constants and the structure of the model (pharmacological values) were found in the literature [28]–[32].

#### Ximelagatran

The binding reactions of melagatran (the active metabolite of ximelagatran) to Factor IIa, meizothrombin (mIIa), and the thrombin–thrombomodulin complex were added into the model (RXi1 to RXi4; Table 2). All kinetic constants and the structure of the model (mechanistic mode of action) were found in the literature [33]–[38].

#### Enoxaparin

The complexation of enoxaparin to AT, and the subsequent reactions of this complex with Factor Xa, the Factor Xa–Factor Va complex (prothrombinase), Factor IIa, mIIa, Factor IXa and Factor XIa were added into the model (Table 2, RHep1 to RHep11). The major inhibitory effect is on Factor Xa (free and lipid bound), which is reflected by the highest rate constant. The model takes into account the dissociation of enoxaparin after any of these targets is bound to activated AT, and enoxaparin is released, allowing it to activate another molecule of AT.

#### Warfarin

The action of warfarin was not modelled explicitly, but represented by a shift in initial conditions (starting concentrations) for vitamin K-dependent Factors II, VII, IX and X, as well as proteins C and S based on published data [39]. To reach a given therapeutic international normalized ratio (INR), each vitamin K-dependent Factor concentration was simultaneously reduced to the required percentage of the normal value.

### Blood flow

**Figure 2:**
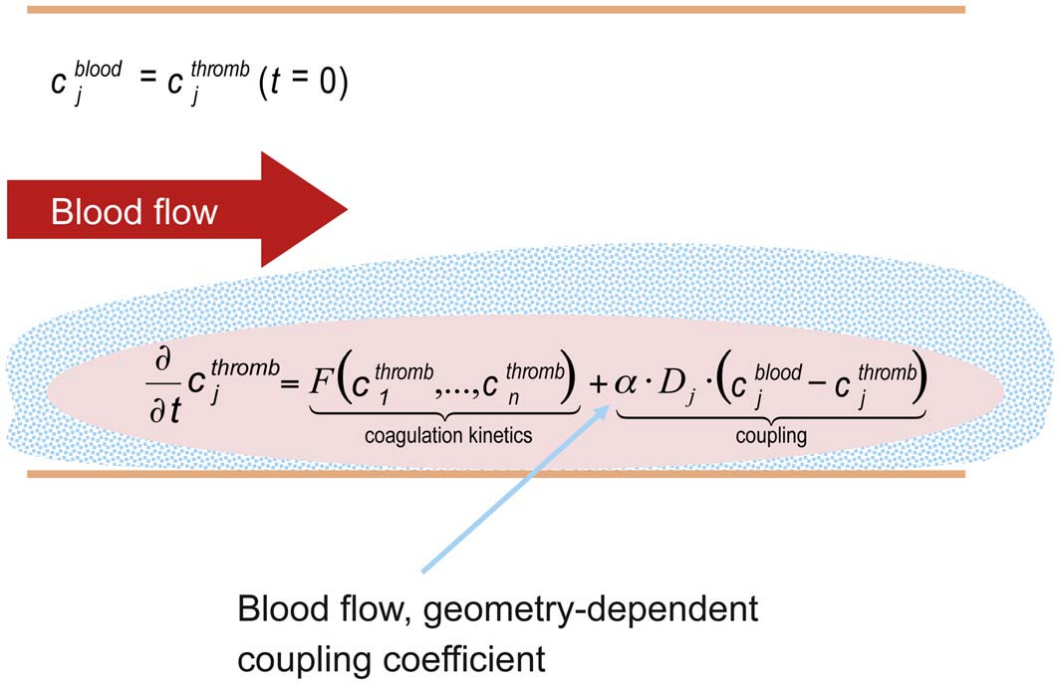
Modelling of blood flow. The top equation represents the initial condition where the concentration of each species c_j_ is the same for the coagulation zone ‘thromb’ and the reservoir ‘blood’. The first element of the bottom equation (‘coagulation kinetics’) represents the coagulation processes (i.e. the biochemical kinetics of the model), whereas the second element (‘coupling’) represents the flowing conditions. α, general coupling constant, as a function of blood flow and geometry (size of vessels); c_j_, concentration of coagulation factor; D_j_, diffusion–convection coefficient; F, blood coagulation cascade model coefficient.

**Figure 3:**
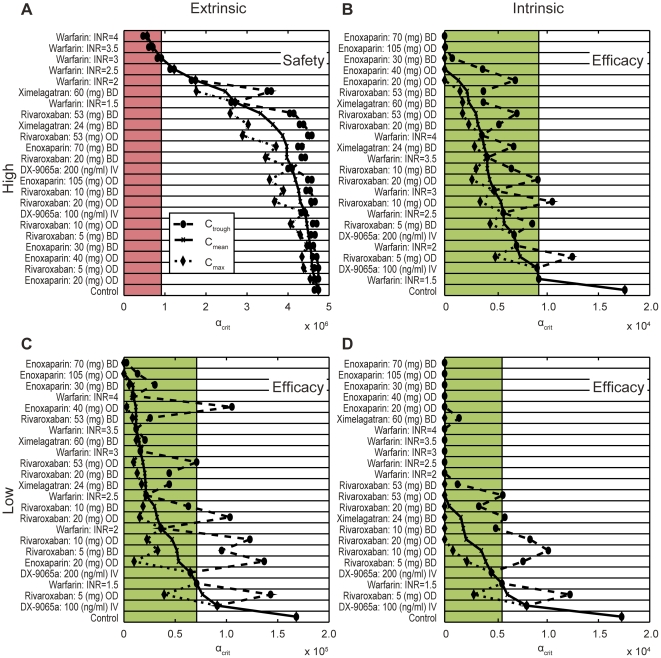
Benchmarking of anticoagulants based on thresholds for blood flow-mediated washout using physiologically plausible trigger concentrations. The dashed line and dots and the dotted line and the diamonds indicate the spread of the threshold for blood flow- and diffusion-mediated washout of a clot formation (α_crit_, seconds; Methods) within the therapeutic concentration range (difference between C_trough_ and C_max_); α_crit_ at C_mean_ is indicated by the solid line and crosses. The strong extrinsic trigger (TF 10^−11^ mol/l, seconds; Table 3) is considered as safety relevant (A). The (red) shaded area indicates safety-relevant prolongation of clotting times above the effect of warfarin titrated to an INR of 3, which is used as a safety reference. All therapies above the level of this therapy are considered safe. The other three triggers (B: Factor XIIa 10^−11^ mol/l; C: TF 10^−14^ mol/l; D: 10^−14^ mol/l) are considered as efficacy relevant. The effect of warfarin titrated to an INR of 1.5 is used as an efficacy reference and all therapies reaching inhibition above the level of this therapy, i.e. α_crit_ values below the reference level (green shaded area) are considered efficacious. BD, twice daily; C_max_, maximum concentration; C_mean_, mean concentration; C_trough_, minimum concentration; INR, international normalized ratio; IV, intravenous; OD, once daily; TF, tissue factor.

The model had to take into account the fact that in vivo coagulation is not only triggered by weak TF and Factor XIIa concentrations and propagated by the coagulation cascade, but is also affected by blood flow and the resulting exchange of proteins between the clotting region (and its surrounding area, named ‘thromb’) and the fresh blood pool that generally includes non-activated coagulation factors (Figure 2). The model described so far was coupled with an infinite pool of plasma (named ‘blood’) to represent the loss of activated factors and the supply of non-activated factors due to blood flow and diffusion (Figure 2). We used an approach similar to Kuharsky and Fogelson [13] and assumed a linear flow parameter, which was identical for all transported species, to describe the mass exchange. This resulted in transport reactions for each species X in the model (see: Appendix S1 and Appendix S2) of the form:
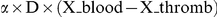



The only species not transported are those bound to immobile tissue or to the thrombus (i.e. TF and all its formed complexes with other species and the fibrin cleavage product Ia). Coupling was determined by a general coupling constant (α), which controls the overall strength of the coupling, and a Factor-dependent diffusion–convection constant, termed D (set to 5×10^−7^ cm^2^/s, averaged from the more complex hydrodynamic approach of Anand et al. (Table 3) [14]. When α is zero, the flow model is equal to the static model; increasing α represents an increase of the blood flow. The lowest exchange rate (i.e. the critical value of α [α_crit_]), which suppresses a coagulation event for a given trigger scenario, was calculated, and comparisons of the different α_crit_ values for the study drugs were a useful model output to benchmark compounds and dosing schedules (Results, Figure 3). However, there were no means to determine α_crit_ directly or correlate it to an experimentally accessible value. Furthermore, it is important to note that this model only focused on the onset of coagulation (i.e. that fibrinolysis was not included in this model).

**Table 3:** *In vivo* coagulation scenarios included in the model.

Scenario	Tissue factor concentration (M)	Factor XIIa concentration (M)
Extrinsic strong	10^−11^	0
Extrinsic weak	10^−14^	0
Intrinsic strong	0	10^−11^
Intrinsic weak	0	10^−14^

### Initial conditions

The initial concentrations for all modelled coagulation factors and other proteins are given in Appendix S2. Depending on the coagulation scenario (*in vitro* or *in vivo*), individual factors not listed in Appendix S2, such as TF or Factor XIIa/Factor XIa (i.e. the triggers), were set to values other than zero, as for the values given in Table 3 or described below for PT and aPTT.

### Kinetic parameters

Kinetic parameters, including kinetic and diffusion constants, are given in Appendix S3. These constants are independent of the simulated coagulation scenario, except for the general flow coupling constant α, the dilution factor (see below), and the albumin–factor coupling constant, which describes the dilution of albumin and affects the unbound fraction of the different study drugs.

### Implementation of coagulation scenarios – *in vitro* scenarios

Dilution of plasma by a coagulation reagent was defined as reduced concentrations of all plasma proteins and factors, as well as added drugs, or as adjusted effective concentrations for surface-bound factors (e.g. TF). Initial plasma concentrations of inhibitors (study drugs) were calculated based on pharmacokinetic data.

#### Prothrombin time

**Figure 4:**
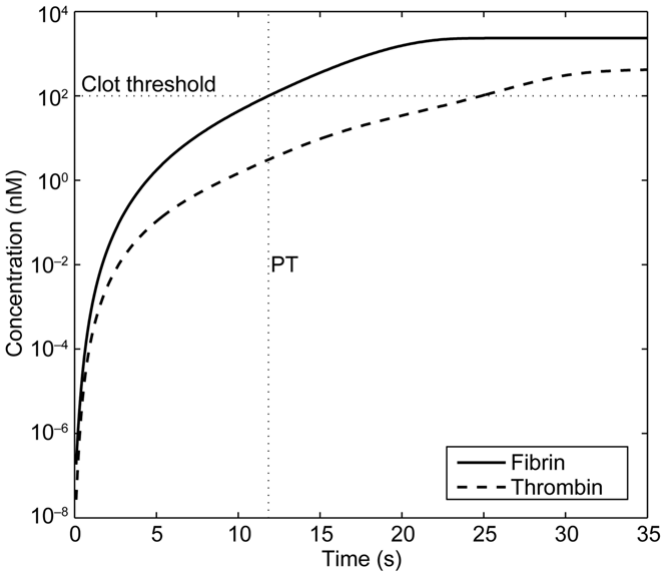
Implementation of PT into *in vitro* coagulation scenarios. Simulation of a PT scenario, thrombin and fibrin generation. PT, prothrombin time.

**Figure 5:**
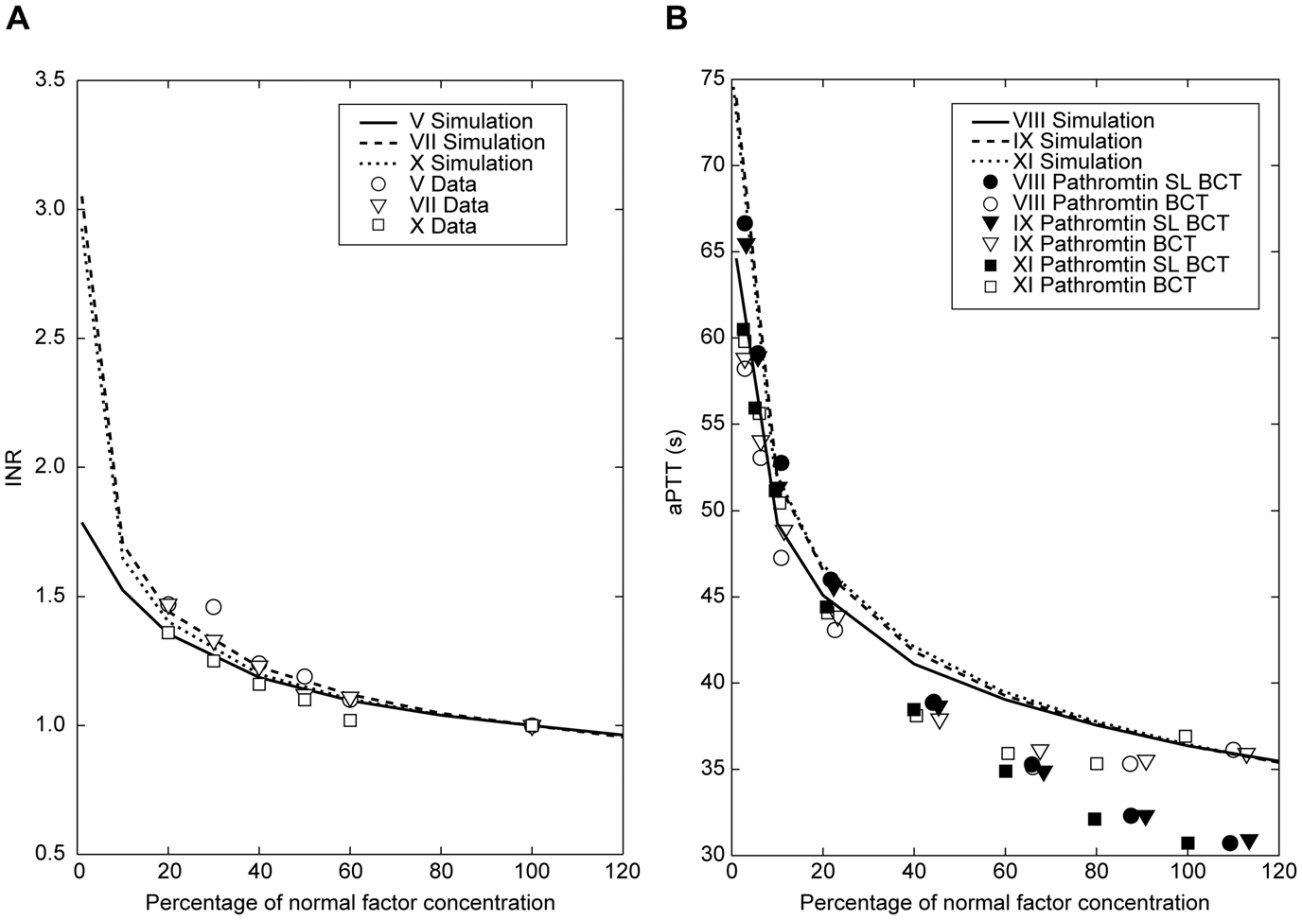
Simulation of coagulation factor variations and comparison with published experimental data. (A) published PT INR data from Fisher Diagnostics 1999 [40] and with (B) published aPTT data from Kappert 2002 [41]. Reagents used in aPTT reference studies: Pathromtin® SL (SL; Behringwerke AG, Marburg, Germany) and Behring Coagulation Time (BCT). aPTT, activated partial thromboplastin time; INR, international normalized ratio; PT, prothrombin time.

All concentrations given in Appendix S2 were reduced to a factor of one-third to account for *in vitro* dilution, in order to reproduce the procedures generally used for the PT test. The tests require that an aliquot of plasma be mixed with an aliquot of coagulation test reagent containing trigger(s), phospholipids, calcium ions and buffer. To initiate a PT test, TF 4 nM concentrations were used, together with preactivated 1% of Factor V taken from [10]. PT was defined as the time when the fibrin concentration reached 100 nM for the first time (Figure 4), meaning that after dilution in the assay, more than 4% of the physiological amount of fibrinogen (7 µM, see Appendix S2) had been activated for clot formation. This was found to be the starting point of a massive cleavage of fibrinogen in the model. The INR was calculated by simply dividing PT with study drug by PT without study drug, thus assuming an ISI exponent of 1 for our simulated PT reagent. The dependence of the INR on individual coagulation factor variance was simulated and compared with published data [40]. The quality of the fit (Figure 5A) was used as validation for the model.

#### Activated partial thromboplastin time

All concentrations given in Appendix S2 were reduced to a factor of one-third to account for *in vitro* dilution and to reproduce the procedures generally used for the aPTT test. To initiate an aPTT test, 2.2 nM Factor XIa and 50 nM Factor XIIa concentrations were used, together with preactivated 1% of Factor V taken from [10]. The coagulation factor variations were simulated and compared with published data [40], [41]. The quality of the fit (Figure 5B) was used as validation for the model.

### Implementation of coagulation scenarios – *in vivo* scenarios

To mimic *in vivo* conditions relevant to bleeding risks under anticoagulant therapy and relevant to thrombosis, four trigger scenarios were constructed (Table 3). All four scenarios are considered to be more closely related to physiological conditions than classical *ex vivo* laboratory tests, where trigger concentrations many orders of magnitude higher than those occurring *in vivo* have to be used for practical reasons.

As a safety-relevant strong trigger, a high TF concentration (10^−11^ mol/l) representing contact with subendothelial tissue was chosen. Prolongation of clotting times under this scenario was interpreted as an increased bleeding risk. To investigate efficacy, three triggers (a low TF concentration, 10^−14^ mol/l, and two Factor XIIa concentrations, 10^−11^ and 10^−14^ mol/l) were separately applied, which represent typical situations where massive clotting should not occur, simulating plasma not in contact with subendothelial tissue. In such scenarios, a significant clotting would raise the risk of thrombosis. These three triggers are interpreted as a complement to the bleeding scenario.

Besides the direct calculation of clotting times under all four trigger scenarios, α_crit_ values were also determined for all these scenarios with and without anticoagulants, the results being used for benchmarking of therapies.

## Results

### Drug action mechanism models of rivaroxaban and other anticoagulants

**Figure 6:**
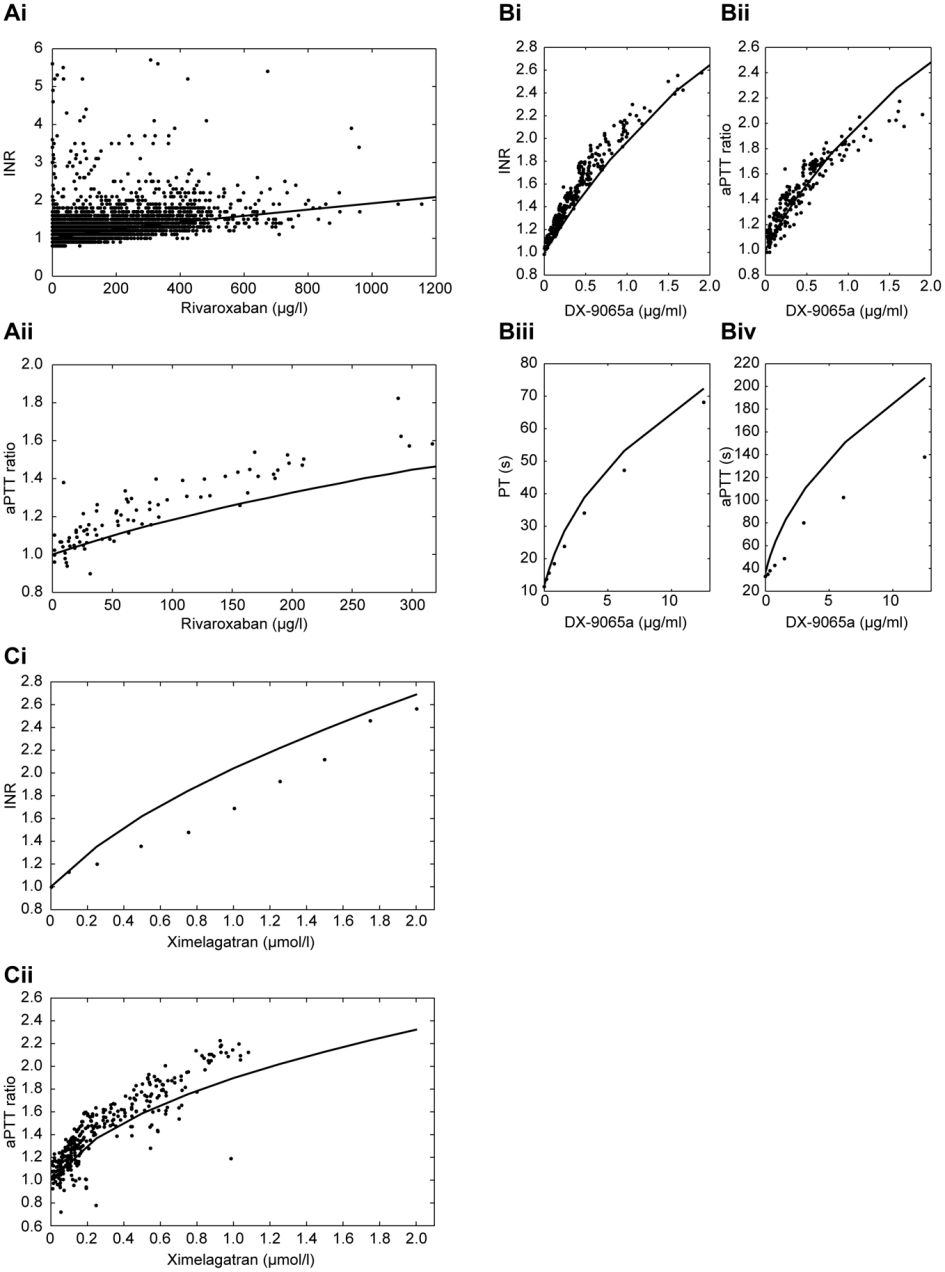
PT and aPTT dependent on plasma concentration of anticoagulant drugs. (A) rivaroxaban (experimental data from internal studies); (B) DX-9065a (experimental data from the literature [30], [31], [41]; and (C) ximelagatran (experimental data for PT and aPTT from the literature [36], [37]. aPTT, activated partial thromboplastin time; INR, international normalized ratio; PT, prothrombin time.

Comparisons were made between experimental concentration–response profiles (data taken from the literature [30], [31], [36], [37], [42] or from in-house studies conducted with rivaroxaban) and simulation results for all study drugs. Plots for PT and aPTT that are dependent on plasma concentrations of rivaroxaban, DX-9065a and ximelagatran in Figures 6A, 6B and 6C, respectively, compare the experimental data with simulated plots for these three drugs [30], [31], [36], [37], [42].

Enoxaparin concentrations in blood are determined in anti-Factor Xa units because it is not a homogenous substance but a mixture of different oligomers [38]. However, using the reported value of 100 units per mg enoxaparin and an average molecular weight of 4500 g/mol, approximate molar concentrations required for modelling can be obtained. PT and aPTT values as a function of plasma concentrations are reported to be not meaningful within the therapeutic concentration range [38], thus being less suited for a model calibration. Anti-Factor Xa values alone on the other hand are not sufficient to validate inactivation kinetics. However, several publications reporting kinetic constants are available [27], [43]–[45]. Averages of these published *in vitro* values were used in our model (Appendix S3, parameters starting with kHep). Simulated PT and aPTT values increased only marginally within therapeutic concentrations (data not shown).

Typical subcutaneous doses are between 20 mg once daily (od) and 30 mg twice daily (bid) for prevention, and 1.5 mg/kg od or 1.0 mg/kg bid for acute therapy. We obtained corresponding plasma concentrations from the manufacturer's data sheets [38] and from the public files of the registration agencies [46]. Unavailable concentrations were taken or extrapolated from in-house measured enoxaparin pharmacokinetic curves. Table 1 lists the concentrations used in our simulations.

**Figure 7:**
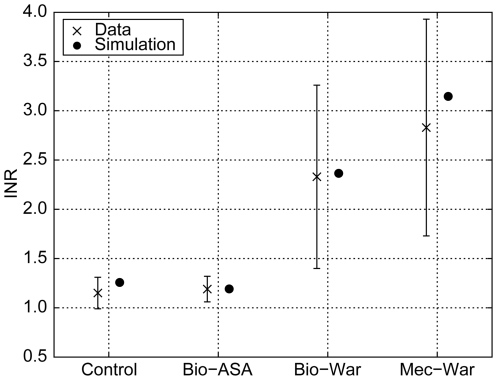
Comparison of simulated with measured INR values. One control group (Control) without medication and three patient groups: mechanical (Mec) or biological (Bio) heart valve replacement with different co-medications (acetylsalicylic acid [ASA] or warfarin [War]) at different target INRs with model predictions of INRs [47]. Simulated INR values were based on measurements of the mean concentrations of Factors II, VII, X, protein C and protein S resulting from therapy. Factor IX was set to the average concentration of the measured factors. INR, international normalized ratio.

Warfarin validation results are shown in Figure 7. The resulting prolongations of the simulated PT values, depicted as INRs, were in good agreement with published data [47]. Variations in experimental data were primarily caused by individual variations in factor concentrations, which are frequent with warfarin therapy, and less by experimental error. Simulations were conducted for the mean concentrations and, therefore, the results presented are close to the actual mean INR data.

### Applications of the model to clinical studies

#### Efficacy and safety compared with warfarin

**Figure 8:**
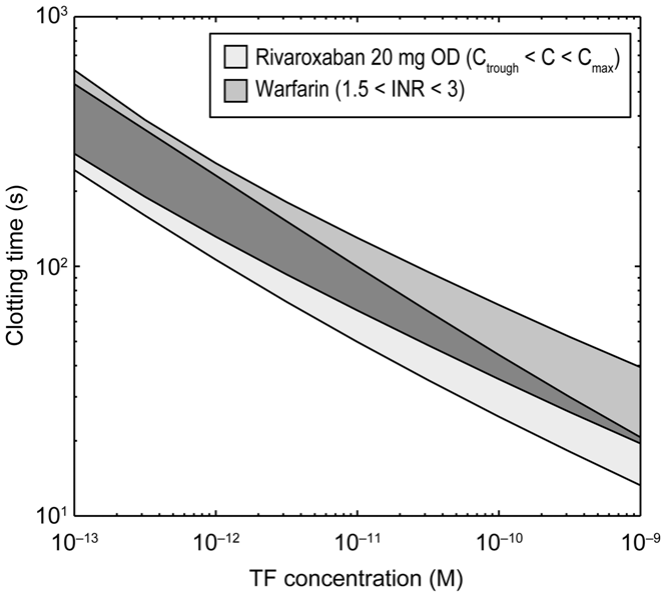
Simulation of thrombus inhibition with rivaroxaban and warfarin. Rivaroxaban concentrations at 20 mg once daily: C_max_ 251 ng/ml, C_trough_ 30 ng/ml. The width of the rivaroxaban curve reflects the C_trough_ to C_max_ concentration range and the width of the warfarin curve reflects the typically used INR range. C_max_, maximum concentration; C_trough_, minimum concentration; INR, international normalized ratio; OD, once daily; TF, tissue factor.

Rivaroxaban 20 mg od was compared with warfarin (INR 1.5–3.0). Clotting times were measured as a function of TF concentrations (Figure 8). At low TF concentrations, the therapeutic INR window for warfarin has a large overlap with the concentration window of the direct Factor Xa inhibitor. By contrast, warfarin shows a stronger effect (i.e. prolongation) on clotting times at higher TF concentrations than the direct Factor Xa inhibitor rivaroxaban. In other words, the mechanism of direct Factor Xa inhibition shows a steeper dependency on TF trigger concentrations than vitamin K-dependent inhibition of the coagulation systems. There is a clear tendency to higher potency at low (i.e. thrombosis relevant) TF concentrations and a lower potency at high TF concentrations where anticoagulant effects could lead to bleeding.

**Figure 9:**
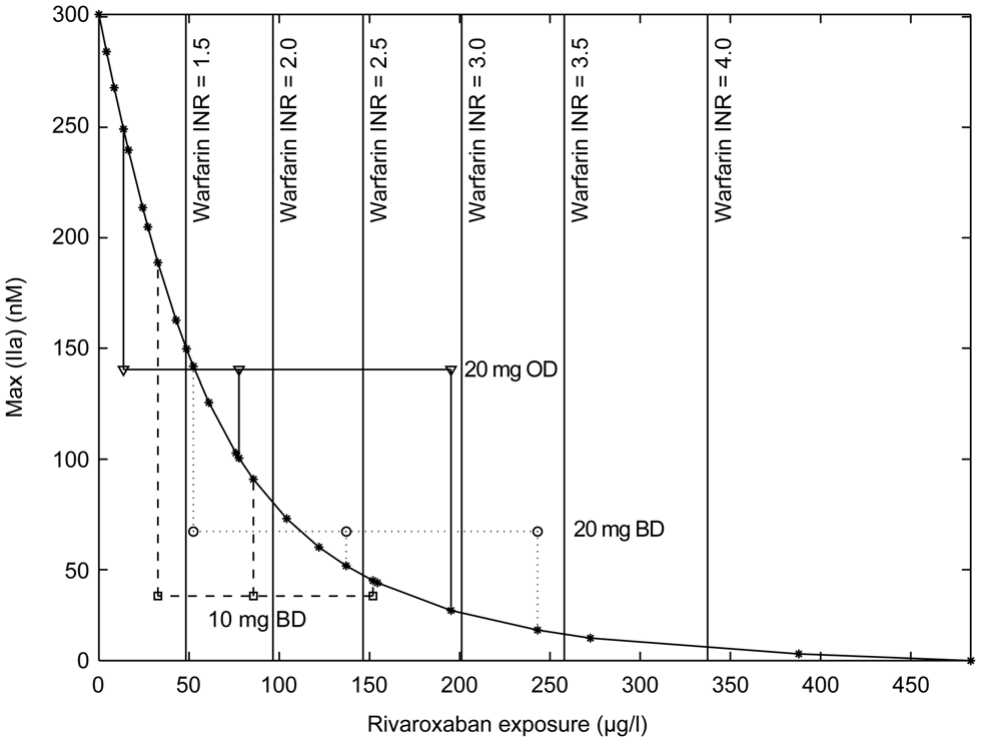
Effect of rivaroxaban exposure on thrombin peak height. This graph shows the effect of increasing concentration of rivaroxaban on thrombin peak height for a 5×10^−12^ mol/l tissue factor trigger (dilution 2∶3, 4 µM phospholipid, corresponding to an *in silico* thrombin generation assay). To compare this with warfarin therapy, the corresponding values for INRs (1.5–4.0) are marked by vertical lines. The ranges for C_max_, C_mean_ and C_trough_ reflect experimental rivaroxaban values found in dose-finding studies [48], [50], [55]. The simulated thrombin peak reduction is in the range of therapeutically used INRs. BD, twice daily; C_max_, maximum concentration; C_mean_, mean concentration; C_trough_, minimum concentration; INR, international normalized ratio; OD, once daily.

Plasma concentrations of a direct Factor Xa inhibitor required to achieve effects similar to warfarin therapy, titrated to different INR values, were then calculated. Figure 9 shows peak concentrations of Factor IIa obtained for a trigger scenario with 5×10^−12^ mol/l TF, (between the value of the extrinsic strong and weak triggers of Table 3), and increasing rivaroxaban concentrations. The concentration–effect curve shows a steep slope at low rivaroxaban concentrations and levels off at concentrations above 150 µg/l. According to this simulated scenario, this type of concentration–effect relationship appears optimal for a broad therapeutic window. Both the under-dosing and over-dosing risks are minimized because low concentrations in the order of the C_trough_ concentrations of Table 1 already reach significant efficacy levels, and variance at high concentrations result in minimal reduction of the thrombin peak.

#### Dose-finding studies

**Figure 10:**
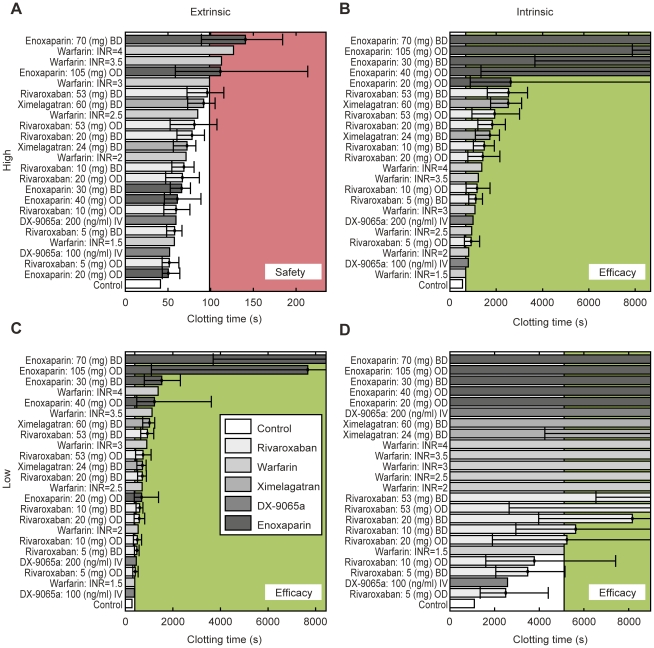
Benchmarking of anticoagulants based on clotting times using physiologically plausible trigger concentrations. Error bars show the spread of the clotting time within the therapeutic concentration range (difference between C_trough_ and C_max_); clotting time at C_mean_ was used for the main bar. The strong extrinsic trigger (TF 10^−11^ mol/l, seconds; Table 3) is considered as safety relevant (A). The (red) shaded area indicates safety-relevant prolongation of clotting times above the effect of warfarin titrated to an INR of 3, which is used as a safety reference. All therapies below the level of this therapy are considered safe. The other three triggers (B: Factor XIIa 10^−11^ mol/l; C: TF 10^−14^ mol/l; D: 10^−14^ mol/l) are considered as efficacy relevant. The effect of warfarin titrated to an INR of 1.5 is used as an efficacy reference and all therapies reaching inhibition above the level of this therapy (green shaded area) are considered efficacious. BD, twice daily; C_max_, maximum concentration; C_mean_, mean concentration; C_trough_, minimum concentration; INR, international normalized ratio; i.v., intravenous; OD, once daily, TF, tissue factor.

The therapeutic window of rivaroxaban was estimated by comparing rivaroxaban with ximelagatran, enoxaparin, DX-9065a and warfarin. The study drugs were simulated and ranked for their effect on clotting time and α_crit_ with all triggers from the panel of physiologically plausible intrinsic and extrinsic activations (Methods, Table 3). Typical doses for all drugs were obtained after reviewing the literature. Figure 10 shows the ranking of clotting times for all scenarios tested. For safety assessment, the warfarin effect at an INR of 3 was used as a reference (upper threshold, red shaded area). For efficacy assessment, the warfarin effect at an INR of 1.5 was used (lower threshold, green shaded area). Using this (relative) measure, all rivaroxaban therapies up to a dose of 20 mg bid are considered safe. Depending on the efficacy scenario, daily doses of rivaroxaban above 5 mg appear to be efficacious, with a daily dose of 20 mg reaching the efficacy window in all three scenarios. Similar results were obtained with the calculation of the α_crit_ levels; daily doses of rivaroxaban between 5 mg and 40 mg appear to be safe and efficacious, with a possible optimal dose of approximately 20 mg per day.

## Discussion

The blood coagulation modelling presented in this paper allows robust simulation of clinically relevant blood coagulation tests and of *in silico* experiments that simulate flowing blood. The effects of coagulation inhibitors acting upon coagulation factors can be easily simulated using the graphical user interface MoBi. In combination with experimental or simulated pharmacokinetic data, therapeutic ranges for the drugs included in the model can be evaluated and efficacy and safety limits can be determined.

The comparative analysis of the mechanism of direct Factor Xa inhibition (rivaroxaban) showed a dependency on TF trigger strength favourable to the vitamin K-dependent inhibition of the coagulation system (Figure 8). At low trigger concentrations assumed to be relevant for thrombosis prevention, the potency of both mechanisms of action is similar, but at high trigger concentrations, relevant for bleeding risk assessment, the effect of direct inhibition is smaller. The concentration–effect curve of rivaroxaban (Figure 9) shows a pronounced convex shape. The increase of inhibition at low inhibitor concentrations is much steeper than at high concentrations. Together with the TF concentration dependency, this result supports the assumption of a broad therapeutic range for direct Factor Xa inhibition.

The therapeutic window for rivaroxaban found by simulating and ranking a broad portfolio of anticoagulants and scenarios was a total daily dose between 5 mg and 40 mg (Figures 3 and 10). Within this window, direct Factor Xa inhibition with rivaroxaban compares well with other currently used or developed anticoagulant therapies and consistently outperforms vitamin K-dependent inhibition (warfarin). A 20 mg od dose yielded favourable coagulation results, i.e. the safety margin of an INR 3 warfarin therapy was not exceeded, and efficacy was better than an INR 1.5 therapy (see Figures 3 and 10). Dose-finding, phase IIb studies of rivaroxaban for the prevention of venous thromboembolism in patients undergoing total hip and knee replacement showed that 5–20 mg total daily doses had efficacy and safety similar to that of enoxaparin, thereby demonstrating a wide therapeutic window for rivaroxaban [48], [49].

After another phase II study investigating the once-daily dosing of rivaroxaban in patients undergoing total hip replacement [50], the 10 mg od dose was considered to provide the optimal balance between efficacy and safety. It was selected for further development in the phase III RECORD programme, which investigated the prevention of venous thromboembolism after total hip or total knee replacement [51]–[54].

After two dose-ranging phase II studies for the treatment of venous thromboembolism [55], [56], a starting dose of 15 mg bid followed by a 20 mg od maintenance dose of rivaroxaban was selected for phase III studies in this indication. These doses are within the therapeutic window of 5–50 mg determined by the present computer model, thereby confirming its usefulness and quantitative accuracy. Potential applications in modelling include other aspects of coagulation, such as medication switch or the combination of antithrombotic therapies (e.g. anticoagulant plus platelet inhibitors) in the therapy of acute coronary syndrome.

## Supporting Information

Appendix S1 Comprehensive reaction list of the coagulation model.
(DOC)

Appendix S2 Initial conditions for all species not being zero (coagulation factors and intrinsic inhibitors).
(DOC)

Appendix S3 Kinetic parameters for the model (based on mol/l and seconds).
(DOC)
