# Supplementary material for: Evaluation of the Efficacy and Safety of Rivaroxaban Using a Computer Model for Blood Coagulation
Source: PLoS One. 2011 Apr 22;6(4):e17626. doi: 10.1371/journal.pone.0017626 (PMC3081290; doi:10.1371/journal.pone.0017626)
Supplement: Appendix S1 — Comprehensive reaction list of the coagulation model. (DOC) [file pone.0017626.s001.doc]

**Supporting Information S1.** Comprehensive reaction list of the coagulation model.

| **Name** | **Stoichiometry** | **Kinetic** |
| --- | --- | --- |
| APCa1AT | APC + alpha1AT -> APC_alpha1AT | Meer1*APC*alpha1AT |
| APCa2AP | APC + alpha2AP -> APC_a2AP | Heeb2*APC*alpha2AP |
| APCa2MG | APC + a2MG -> APC_a2MG | Heeb1*APC*a2MG |
| APCPCI | APC + PCI -> APC_PCI | Herm4*APC*PCI-Herm4*Herm4i*APC_PCI |
| APCPNI | APC + PNI -> APC_PNI | Herm2*APC*PNI-Herm2*Herm2i*APC_PNI |
| mTmAT | mIIa_Tm + ATIII -> mIIa_ATIII + Tm | mIIaFactor1*Bourin1*mIIa_Tm*ATIII |
| mTmPCI | mIIa_Tm + PCI -> mIIa_PCI + Tm | Elisen1*mIIa_Tm*PCI*mIIaFactor1 |
| R1 | TF + VII -> TF_VII | k2*TF*VII-k1*TF_VII |
| R10 | VIII_lipid -> VIIIa_lipid | (k17*IIa+k17b*mIIa+k17c*(Xa_lipid+Xa))*VIII_lipid |
| R10d | VIII -> VIIIa | (k17*IIa+k17b*mIIa+k17c*(Xa_lipid+Xa))*VIII |
| R10vWF | VIII_vWF -> VIIIa_vWF | k17vWF*(IIa+mIIa)*VIII_vWF |
| R11 | VIIIa_lipid + IXa_lipid -> IXa_VIIIa_lipid + Phospholipid | k19*VIIIa_lipid*IXa_lipid-k18*IXa_VIIIa_lipid |
| R12 | IXa_VIIIa_lipid + X_lipid ­> IXa_VIIIa_X_lipid + Phospholipid | k21*IXa_VIIIa_lipid*X_lipid-k20*IXa_VIIIa_X_lipid |
| R12a | IXa_VIIIa_X_lipid + Phospholipid -> IXa_VIIIa_lipid + Xa_lipid | k22*IXa_VIIIa_X_lipid |
| R13 | VIIIa_lipid -> VIIIa1_L + VIIIa2 + Phospholipid | k24*VIIIa_lipid-k23*VIIIa1_L*VIIIa2 |
| R14 | IXa_VIIIa_X_lipid + Phospholipid -> VIIIa1_L + VIIIa2 + X_lipid + IXa_lipid | k25*IXa_VIIIa_X_lipid |
| R15 | IXa_VIIIa_lipid -> VIIIa1_L + VIIIa2 + IXa_lipid | k25*IXa_VIIIa_lipid |
| R16 | V_lipid -> Va_lipid | (k26*IIa+k26b*mIIa+k26c*(Xa_lipid+Xa))*V_lipid |
| R16d | V -> Va | (k26*IIa+k26b*mIIa+k26c*(Xa+Xa_lipid))*V |
| R17d | Xa + Va_lipid -> Xa_Va_lipid | k28*Xa*Va_lipid-k27*Xa_Va_lipid |
| R18 | Xa_Va_lipid + II_lipid -> Xa_Va_II_lipid + Phospholipid | k30*Xa_Va_lipid*II_lipid |
| R18a | Xa_Va_II_lipid -> Xa_Va_lipid + mIIa | k31*Xa_Va_II_lipid |
| R18d | Xa_Va_lipid + II -> Xa_Va_II_lipid | k30*Xa_Va_lipid*II-k29*Xa_Va_II_lipid |
| R19 | mIIa -> IIa | k32*mIIa*Xa_Va_lipid |
| R2 | TF + VIIa -> TF_VIIa | k4*TF*VIIa-k3*TF_VIIa |
| R20 | Xa + TFPI -> Xa_TFPI | k34*Xa*TFPI-k33*Xa_TFPI |
| R21 | TF_VIIa_Xa + TFPI -> TF_VIIa_Xa_TFPI | k36*TF_VIIa_Xa*TFPI-k35*TF_VIIa_Xa_TFPI |
| R22 | TF_VIIa + Xa_TFPI -> TF_VIIa_Xa_TFPI | k37*TF_VIIa*Xa_TFPI |
| R23 | Xa + ATIII -> Xa_ATIII | k38*Xa*ATIII |
| R23a2MG | Xa + a2MG -> Xa_a2MG | Ellis1*Xa*a2MG |
| R23APC | APC + ATIII -> APC_ATIII | Herm5*APC*ATIII |
| R23PCI | Xa + PCI -> Xa_PCI | Suzu1*Xa*PCI-Suzu1*Suzu1i*Xa_PCI |
| R24 | mIIa + ATIII -> mIIa_ATIII | k39*mIIa*ATIII |
| R24a2MG | mIIa + a2MG -> mIIa_a2MG | Down1*mIIa*a2MG |
| R24alpha1AT | mIIa + alpha1AT -> mIIa_alpha1AT | Down2*mIIa*alpha1AT |
| R24HCII | mIIa + HCII -> mIIa_HCII | Veer1*mIIa*HCII |
| R24PCI | mIIa + PCI -> mIIa_PCI | Herm3*mIIa*PCI-Herm3*Herm3i*mIIa_PCI |
| R24PNI | mIIa + PNI -> mIIa_PNI | Herm1*mIIa*PNI-Herm1*Herm1i*mIIa_PNI |
| R25 | IXa + ATIII -> IXa_ATIII | k40*IXa*ATIII |
| R26 | IIa + ATIII -> IIa_ATIII | k41*IIa*ATIII |
| R26a2MG | IIa + a2MG -> IIa_a2MG | Down1*IIa*a2MG |
| R26alpha1AT | IIa + alpha1AT -> IIa_alpha1AT | Down2*IIa*alpha1AT |
| R26HCII | IIa + HCII -> IIa_HCII | Veer1*IIa*HCII |
| R26PCI | IIa + PCI -> IIa_PCI | Herm3*IIa*PCI-Herm3*Herm3i*IIa_PCI |
| R26PNI | IIa + PNI -> IIa_PNI | Herm1*IIa*PNI-Herm1*Herm1i*IIa_PNI |
| R27 | TF_VIIa + ATIII -> TF_VIIa_ATIII | k42*TF_VIIa*ATIII |
| R3 | TF_VIIa + VII -> TF_VIIa + VIIa | k5*TF_VIIa*VII |
| R4 | VII -> VIIa | k6*(Xa+Xa_lipid)*VII |
| R5 | VII -> VIIa | k7*IIa*VII |
| R6 | TF_VIIa + X -> TF_VIIa_X | k9*TF_VIIa*X-k8*TF_VIIa_X |
| R6a | TF_VIIa_X -> TF_VIIa_Xa | k10*TF_VIIa_X |
| R6s | TF_VIIa + X_lipid -> TF_VIIa_X + Phospholipid | k9*TF_VIIa*X_lipid-k8*TF_VIIa_X |
| R7 | TF_VIIa + Xa -> TF_VIIa_Xa | k12*TF_VIIa*Xa-k11*TF_VIIa_Xa |
| R7s | TF_VIIa + Xa_lipid -> TF_VIIa_Xa + Phospholipid | k12*TF_VIIa*Xa_lipid-k11*TF_VIIa_Xa |
| R8 | TF_VIIa + IX -> TF_VIIa_IX | k14*TF_VIIa*IX-k13*TF_VIIa_IX |
| R8a | TF_VIIa_IX -> TF_VIIa + IXa | k15*TF_VIIa_IX |
| R8as | TF_VIIa_IX_lipid -> TF_VIIa + IXa_lipid | k15*TF_VIIa_IX_lipid |
| R8s | TF_VIIa + IX_lipid -> TF_VIIa_IX_lipid | k14*TF_VIIa*IX_lipid-k13*TF_VIIa_IX_lipid |
| R9 | II -> IIa | k16*(Xa_lipid+Xa)*II |
| R9s | II_lipid -> IIa + Phospholipid | k16*(Xa_lipid+Xa)*II_lipid |
| Rad19 | IX + Phospholipid -> IX_lipid | k9on*IX*Phospholipid-Kd9*k9on*IX_lipid |
| Rad20 | IXa + Phospholipid -> IXa_lipid | k9on*IXa*Phospholipid-Kd9*k9on*IXa_lipid |
| Rad22 | X + Phospholipid -> X_lipid | k10on*X*Phospholipid-Kd10*k10on*X_lipid |
| Rad23 | Xa + Phospholipid -> Xa_lipid | k10on*Xa*Phospholipid-Kd10*k10on*Xa_lipid |
| Rad24 | V + Phospholipid -> V_lipid | k5on*V*Phospholipid-Kd5*k5on*V_lipid |
| Rad25 | Va + Phospholipid -> Va_lipid | k5on*Va*Phospholipid-Kd5*k5on*Va_lipid |
| Rad26 | VIII + Phospholipid -> VIII_lipid | k8on*VIII*Phospholipid-Kd8*k8on*VIII_lipid |
| Rad27 | VIIIa + Phospholipid -> VIIIa_lipid | k8on*VIIIa*Phospholipid-Kd8*k8on*VIIIa_lipid |
| Rad28 | II + Phospholipid -> II_lipid | k2on*II*Phospholipid-Kd2*k2on*II_lipid |
| Rad31 | XIa + Phospholipid -> XIa_lipid | k11aon*XIa*Phospholipid-Kd11a*k11aon*XIa_lipid |
| Rad32 | Bay59-7939_Xa + Phospholipid -> Bay59­7939_Xa_lipid | k10on*Bay59-7939_Xa*Phospholipid-Kd10*k10on*Bay59-7939_Xa_lipid |
| Rad33 | Dx9065a_Xa + Phospholipid -> Dx9065a_Xa_lipid | k10on*Dx9065a_Xa*Phospholipid-Kd10*k10on*Dx9065a_Xa_lipid |
| Rad34 | XI + Phospholipid -> XI_lipid | k11on*XI*Phospholipid-Kd11*k11on*XI_lipid |
| RBay1 | Bay59-7939 + Xa -> Bay59-7939_Xa | Bay59-7939*Xa*kBay1-Bay59­7939_Xa*kBay1*kBay_Ki_Xa |
| RBay1s | Bay59-7939 + Xa_lipid -> Bay59-7939_Xa_lipid | Bay59-7939*Xa_lipid*kBay1-Bay59­7939_Xa_lipid*kBay1*kBay_Ki_Xa |
| RBay2 | Bay59-7939 + Xa_Va_lipid -> Bay59­7939_Xa_Va_lipid | Bay59-7939*Xa_Va_lipid*kBay3-Bay59­7939_Xa_Va_lipid*kBay3*kBay_Ki_XaVa |
| RBay3 | Bay59-7939_Bound -> Bay59-7939 | kBay_fu_on*kBay_fu*Bay59-7939_Bound-kBay_fu_on*Albumin_Factor*Bay59-7939 |
| RBay4 | Bay59-7939_Xa + ATIII ׁ­> Bay59-7939_Xa_ATIII | kBay5*Bay59-7939_Xa*ATIII |
| RBay5 | Bay59-7939 + Xa_ATIII ­> Bay59-7939_Xa_ATIII | kBay6*Bay59-7939*Xa_ATIII-kBay6*kBay_Ki_XaATIII*Bay59-7939_Xa_ATIII |
| RBu28 | IIa_Tm + PC -> IIa_Tm_PC | kbu43*IIa_Tm*PC-kbu52*IIa_Tm_PC |
| RBu280 | IIa + Tm -> IIa_Tm | kbu50*IIa*Tm-kbu51*IIa_Tm |
| RBu280m | mIIa + Tm -> mIIa_Tm | kbu50m*mIIa*Tm-kbu51*mIIa_Tm |
| RBu28m | mIIa_Tm + PC -> mIIa_Tm_PC | kbu43*mIIa_Tm*PC-kbu52*mIIa_Tm_PC |
| RBu29 | APC + PS -> APC_PS | kbu44*APC*PS-kbu45*APC_PS |
| RBu290 | IIa_Tm_PC -> IIa_Tm + APC | kbu53*IIa_Tm_PC |
| RBu290m | mIIa_Tm_PC -> APC + mIIa_Tm | mIIaFactor2*kbu53*mIIa_Tm_PC |
| RBu30 | APC_PS + VIIIa_lipid -> APC_PS_VIIIa_lipid | kbu46*APC_PS*VIIIa_lipid-kbu54*APC_PS_VIIIa_lipid |
| RBu30b | APC_PS + VIIIa -> APC_PS_VIIIa | kbu46b*APC_PS*VIIIa-kbu54*APC_PS_VIIIa |
| RBu31 | APC_PS + Va_lipid -> APC_PS_Va_lipid | kbu47*APC_PS*Va_lipid-kbu55*APC_PS_Va_lipid |
| RBu31b | APC_PS + Va -> APC_PS_Va | kbu47b*APC_PS*Va-kbu55*APC_PS_Va |
| RBu32 | APC_PS_Va_lipid -> APC_PS + Va_Inhib + Phospholipid | kbu48*APC_PS_Va_lipid |
| RBu32b | APC_PS_Va -> Va_Inhib + APC_PS | kbu48*APC_PS_Va |
| RBu33 | APC_PS_VIIIa_lipid -> APC_PS + VIIIa_Inhib + Phospholipid | kbu49*APC_PS_VIIIa_lipid |
| RBu33b | APC_PS_VIIIa -> VIIIa_Inhib + APC_PS | kbu49*APC_PS_VIIIa |
| RDx1 | Dx9065a + Xa -> Dx9065a_Xa | kDx1*Dx9065a*Xa-kDx2*Dx9065a_Xa |
| RDx1s | Dx9065a + Xa_lipid -> Dx9065a_Xa_lipid | kDx1*Dx9065a*Xa_lipid-kDx2*Dx9065a_Xa_lipid |
| RDx2 | Dx9065a + Xa_Va_lipid ­> Dx9065a_Xa_Va_lipid | kDx3*Dx9065a*Xa_Va_lipid-kDx4*Dx9065a_Xa_Va_lipid |
| RDx3 | Dx9065a_Xa + ATIII -> Dx9065a_Xa_ATIII | kDx5*Dx9065a_Xa*ATIII |
| RDx4 | Dx9065a -> Dx9065a_Bound | kDx_fu_on*Albumin_Factor*Dx9065a-kDx_fu_on*kDx_fu*Dx9065a_Bound |
| RDx5 | Dx9065a + Xa_ATIII -> Dx9065a_Xa_ATIII | kDx6*Dx9065a*Xa_ATIII-kDx7*Dx9065a_Xa_ATIII |
| RE27 | Xa + alpha1AT -> Xa_alpha1AT | Ellis2*alpha1AT*Xa |
| RHep1 | Hep -> Hep_Bound | kHep_fu_on*Albumin_Factor*Hep-kHep_fu_on*kHep_fu*Hep_Bound |
| RHep10 | XIa + ATIIIa -> XIa_ATIII + Hep | kHep_XIa_ATIIIa*XIa*ATIIIa |
| RHep11 | XIa_lipid + ATIIIa -> XIa_ATIII + Phospholipid + Hep | kHep_XIa_ATIIIa*XIa_lipid*ATIIIa |
| RHep2 | ATIII + Hep -> ATIIIa | kHep_ATIII_on*ATIII*Hep-kHep_ATIII_on*kHep_Ki_ATIII*ATIIIa |
| RHep3 | Xa + ATIIIa -> Xa_ATIII + Hep | kHep_Xa_ATIIIa*Xa*ATIIIa |
| RHep4 | Xa_lipid + ATIIIa -> Xa_ATIII + Phospholipid + Hep | kHep_Xa_ATIIIa*Xa_lipid*ATIIIa |
| RHep5 | Xa_Va_lipid + ATIIIa -> Xa_ATIII + Hep + Va_lipid | kHep_XaVa_ATIIIa*Xa_Va_lipid*ATIIIa |
| RHep6 | IIa + ATIIIa -> IIa_ATIII + Hep | kHep_IIa_ATIIIa*IIa*ATIIIa |
| RHep7 | mIIa + ATIIIa -> mIIa_ATIII + Hep | kHep_IIa_ATIIIa*mIIa*ATIIIa |
| RHep8 | ATIIIa + IXa -> IXa_ATIII + Hep | kHep_IXa_ATIIIa*IXa*ATIIIa |
| RHep9 | ATIIIa + IXa_lipid -> IXa_ATIII + Phospholipid + Hep | kHep_IXa_ATIIIa*IXa_lipid*ATIIIa |
| RI | I -> Ia | kI*(IIa+0.07*mIIa)*I/(KIm+I) |
| RIXa | IX_lipid -> IXa_lipid | Kog8*XIa_lipid*IX_lipid/(Kog8m+IX_lipid) |
| RIXad | IX -> IXa | Kog8*XIa*IX/(Kog8m+IX) |
| RK26 | XIa + C1Inh -> XIa_C1Inh | Kog26*C1Inh*XIa |
| RK27 | XIa + alpha1AT -> XIa_alpha1AT | Kog27*alpha1AT*XIa |
| RK28 | XIa + ATIII -> XIa_ATIII | Kog28*ATIII*XIa |
| RK29 | XIa + alpha2AP -> XIa_alpha2AP | Kog29*XIa*alpha2AP |
| RK30 | XIa + PAI1 -> XIa_PAI1 | Kog30*XIa*PAI1 |
| RvWF1 | vWF + VIII -> VIII_vWF | vWF*VIII*kvWF1-VIII_vWF*kvWF2 |
| RvWF2 | vWF + VIIIa -> VIIIa_vWF | vWF*VIIIa*kvWF3-VIIIa_vWF*kvWF4 |
| RXa | X_lipid -> Xa_lipid | Kog9*IXa_lipid*X_lipid/(Kog9m+X_lipid) |
| RXi1 | Xim + IIa -> IIa_Xim | kXim1*IIa*Xim-kXim2*IIa_Xim |
| RXi2 | Xim + mIIa -> mIIa_Xim | kXim3*mIIa*Xim-kXim4*mIIa_Xim |
| RXi3 | Xim + IIa_Tm -> IIa_Tm_Xim | kXim5*IIa_Tm*Xim-kXim6*IIa_Tm_Xim |
| RXi4 | Xim -> Xim_Bound | kXim_fu_on*Albumin_Factor*Xim-kXim_fu_on*kXim_fu*Xim_Bound |
| RXIaII | XI -> XIa | Kog10*(IIa+mIIa)*XI/(Kog10m+XI) |
| RXIaIIs | XI_lipid -> XIa_lipid | Kog10*(IIa+mIIa)*XI_lipid/(Kog10m+XI_lipid) |
| RXIaXII | XI -> XIa | Kog6*XIIa*XI/(Kog6m+XI) |
| RXIaXIIs | XI_lipid -> XIa_lipid | Kog6*XIIa*XI_lipid/(Kog6m+XI_lipid) |
| TmAT | IIa_Tm + ATIII -> Tm + IIa_ATIII | Bourin1*IIa_Tm*ATIII |
| TmPCI | IIa_Tm + PCI -> Tm + IIa_PCI | Elisen1*IIa_Tm*PCI |

IIa, Va, VIIa, VIIIa, IXa, Xa, XIa denote activated coagulation factors. a2MG, α2-macroglobulin; alpha1AT, alpha1-antitrypsin; alpha2AP, alpha2-antiplasmin; ATIII, antithrombin; Bay59-7939, rivaroxaban; C1Inh, C1 inhibitor; fu, fraction unbound; Hep, heparin (here parameterized as enoxaparin); HCII, heparin cofactor II; PAI1, plasminogen activator inhibitor; PC, protein C; PCI, protein C inhibitor; PNI, protease nexin I; PS, protein S; TFPI, tissue factor pathway inhibitor; Tm, thrombomodulin; vWF, von Willebrand factor; Xim, ximelagatran active metabolite (melagatran).
