# Supplementary material for: Evaluation of the Efficacy and Safety of Rivaroxaban Using a Computer Model for Blood Coagulation
Source: PLoS One. 2011 Apr 22;6(4):e17626. doi: 10.1371/journal.pone.0017626 (PMC3081290; doi:10.1371/journal.pone.0017626)
Supplement: Appendix S2 — Initial conditions for all species not being zero (coagulation factors and intrinsic inhibitors). (DOC) [file pone.0017626.s002.doc]

**Supporting Information S2.** Initial conditions for all species not being zero (coagulation factors and intrinsic inhibitors).

| **Species** | **Full Name** | **Initial (mol/l)** |
| --- | --- | --- |
| a2MG | α_2_-Macroglobulin | 3.11E-06 |
| alpha1AT | α _1_-Antitrypsin | 2.45E-05 |
| alpha2AP | α_2_-Antiplasmin | 9.00E-07 |
| ATIII | Antithrombin | 2.50E-06 |
| C1Inh | C1 inhibitor | 1.70E-06 |
| HCII | Heparin cofactor II | 1.20E-06 |
| I | Fibrinogen | 7.00E-06 |
| II | Thrombin (Factor II) | 1.40E-06 |
| IX | Factor IX | 9.00E-08 |
| PAI1 | Plasminogen activator inhibitor | 4.60E-10 |
| PC | Protein C | 6.00E-08 |
| PCI | Protein C inhibitor | 8.80E-08 |
| PNI | Protease nexin I | 2.00E-11 |
| PS | Protein S | 1.40E-07 |
| TFPI | Tissue factor pathway inhibitor | 2.50E-09 |
| Tm | Thrombomodulin | 3.30E-10 |
| V | Factor V | 2.00E-08 |
| VII | Factor VII | 1.00E-08 |
| VIIa | Factor VIIa | 1.00E-10 |
| VIII | Factor VIII | 7.00E-10 |
| vWF | von Willebrand factor | 3.50E-08 |
| X | Factor X | 1.60E-07 |
| Xa | Factor Xa | 1.60E-14 |
| XI | Factor XI | 2.50E-08 |
