# Supplementary material for: Evaluation of the Efficacy and Safety of Rivaroxaban Using a Computer Model for Blood Coagulation
Source: PLoS One. 2011 Apr 22;6(4):e17626. doi: 10.1371/journal.pone.0017626 (PMC3081290; doi:10.1371/journal.pone.0017626)
Supplement: Appendix S3 — Kinetic parameters for the model (based on mol/l and seconds). (DOC) [file pone.0017626.s003.doc]

**Supporting Information S3.** Kinetic parameters for the model (based on mol/l and seconds).

| **Name** | **Value** | **Name** | **Value** | **Name** | **Value** |
| --- | --- | --- | --- | --- | --- |
| Albumin_Factor | 1 | k36 | 2.72E+08 | kHep_ATIII_on | 5.94E+07 |
| Bourin1 | 31950 | k37 | 5.00E+07 | kHep_fu | 19 |
| DilutionFactor | 1 | k38 | 1500 | kHep_fu_on | 0.53 |
| Down1 | 488.333 | k39 | 13995,167 | kHep_IIa_ATIIIa | 5.50E+05 |
| Down2 | 108.5 | k4 | 2.30E+07 | kHep_IXa_ATIIIa | 10000 |
| Elisen1 | 25212.396 | k40 | 490 | kHep_Ki_ATIII | 7.70E-06 |
| Ellis1 | 718 | k41 | 280 | kHep_Xa_ATIIIa | 1.91E+06 |
| Ellis2 | 226.667 | k42 | 230 | kHep_XaVa_ATIIIa | 5.70E+05 |
| Heeb1 | 86 | k5 | 440000 | kHep_XIa_ATIIIa | 150 |
| Heeb2 | 100 | k5on | 1.50E+06 | kI | 528.42 |
| HepActivityFactor | 1 | k6 | 1.30E+07 | KIm | 3.16E-06 |
| Herm1 | 1.48E+06 | k7 | 23000 | Kog1 | 0.033 |
| Herm1i | 2.70E-12 | k8 | 1.05 | Kog10 | 0.017 |
| Herm2 | 5200 | k8on | 5.00E+07 | Kog10m | 4.66E-10 |
| Herm2i | 8.00E-10 | k9 | 2.50E+07 | Kog1m | 1.10E-05 |
| Herm3 | 33100 | k9on | 5.00E+07 | Kog2 | 3.6 |
| Herm3i | 1.10E-09 | kBay_fu | 0.054 | Kog26 | 16.667 |
| Herm4 | 800 | kBay_fu_on | 0.175 | Kog27 | 66.6 |
| Herm4i | 1.19E-08 | kBay_Ki_Xa | 4.00E-10 | Kog28 | 166.6 |
| Herm5 | 0.148 | kBay_Ki_XaATIII | 4.00E-10 | Kog29 | 500 |
| k1 | 3.10E-03 | kBay_Ki_XaVa | 4.00E-10 | Kog2m | 9.10E-08 |
| k10 | 6 | kBay1 | 5.40E+07 | Kog3 | 40 |
| k10on | 1.00E+07 | kBay3 | 1.50E+06 | Kog30 | 210000 |
| k11 | 19 | kBay5 | 0 | Kog3m | 3.70E-05 |
| k11aon | 1.00E+07 | kBay6 | 0 | Kog4 | 5.7 |
| k11on | 1.00E+07 | kbu_Factor1 | 3.44E-03 | Kog4m | 5.10E-07 |
| k12 | 2.20E+07 | kbu43 | 1000000 | Kog5 | 5.67E-03 |
| k13 | 2.4 | kbu44 | 1000000 | Kog5m | 5.00E-07 |
| k14 | 1.00E+07 | kbu45 | 5.00E-03 | Kog6 | 0.11 |
| k15 | 0.3 | kbu46 | 1.00E+07 | Kog6m | 2.00E-06 |
| k16 | 62683.558 | kbu46b | 34354.671 | Kog7 | 0.567 |
| k17 | 6.50E+06 | kbu47 | 1.57E+08 | Kog7m | 5.00E-07 |
| k17b | 6.50E+06 | kbu47b | 537739.141 | Kog8 | 0.238 |
| k17c | 1.10E+06 | kbu48 | 0.8 | Kog8m | 3.50E-07 |
| k17vWF | 36000 | kbu49 | 0.8 | Kog9 | 3.33E-04 |
| k18 | 5.00E-03 | kbu50 | 236643.283 | Kog9m | 2.00E-06 |
| k19 | 5.90E+07 | kbu50m | 236643.283 | kvWF1 | 640000 |
| k2 | 160000 | kbu51 | 2.50E-03 | kvWF2 | 2.30E-04 |
| k20 | 1.00E-03 | kbu52 | 0.064 | kvWF3 | 59000 |
| k21 | 1.14E+09 | kbu53 | 3.6 | kvWF4 | 0.038 |
| k22 | 99.56 | kbu54 | 0.16 | kXim_fu | 9 |
| k23 | 22000 | kbu55 | 0.16 | kXim_fu_on | 0.53 |
| k24 | 6.00E-03 | Kd10 | 2.30E-07 | kXim1 | 1.20E+07 |
| k25 | 1.00E-03 | Kd11 | 1.00E-08 | kXim2 | 0.036 |
| k26 | 2.79E+06 | Kd11a | 1.70E-09 | kXim3 | 1.20E+07 |
| k26b | 5.02E+06 | Kd2 | 2.30E-07 | kXim4 | 0.036 |
| k26c | 46000.009 | Kd5 | 1.00E-09 | kXim5 | 2.71E+07 |
| k27 | 0.2 | Kd8 | 2.50E-09 | kXim6 | 0.036 |
| k28 | 2.51E+09 | Kd9 | 2.50E-09 | Meer1 | 10.033 |
| k29 | 103 | kDx_fu | 0.538 | Suzu1 | 9833.333 |
| k2on | 1.00E+07 | kDx_fu_on | 0.53 | Suzu1i | 3.10E-07 |
| k3 | 3.10E-03 | kDx1 | 5.00E+06 | Veer1 | 2000 |
| k30 | 1.00E+08 | kDx2 | 0.205 |  |  |
| k31 | 99.04 | kDx3 | 5.00E+06 |  |  |
| k32 | 5.30E+07 | kDx4 | 0.205 |  |  |
| k33 | 3.60E-04 | kDx5 | 1500 |  |  |
| k34 | 900000 | kDx6 | 5.00E+06 |  |  |
| k35 | 7.27E-03 | kDx7 | 0.205 |  |  |

IIa, Va, IXa, Xa, XIa denote activated coagulation factors. The names of kinetic parameters refer to their sources, e.g. kbu(number) has been derived from (9), k(number) from (10), Kog(number) from (11). ATIII, antithrombin; fu, fraction unbound; Hep, heparin (here parameterized as enoxaparin); vWF, von Willebrand factor; Xim, ximelagatran active metabolite (melagatran).
